# Rates, Indications, and Speech Perception Outcomes of Revision Cochlear Implantations

**DOI:** 10.3390/jcm10153215

**Published:** 2021-07-21

**Authors:** Doron Sagiv, Yifat Yaar-Soffer, Ziva Yakir, Yael Henkin, Yisgav Shapira

**Affiliations:** 1 Department of Otolaryngology—Head and Neck Surgery, Sheba Medical Center, Tel Hashomer 5262100, Israel; yisgav.shapira@sheba.health.gov.il; 2 Sackler Faculty of Medicine, Tel Aviv University, Tel Aviv City 6997801, Israel; 3 Hearing, Speech, and Language Center, Sheba Medical Center, Tel Hashomer 5262100, Israel; yifat.yaarsoffer@sheba.health.gov.il (Y.Y.-S.); Ziva.Yakir@sheba.health.gov.il (Z.Y.); henkin@tauex.tau.ac.il (Y.H.); 4 Department of Communication Disorders, Sackler Faculty of Medicine, Tel Aviv University, Tel Aviv City 6997801, Israel

**Keywords:** cochlear implant, revision cochlear implant, speech perception, device failure, soft failure, hard failure

## Abstract

Revision cochlear implant (RCI) is a growing burden on cochlear implant programs. While reports on RCI rate are frequent, outcome measures are limited. The objectives of the current study were to: (1) evaluate RCI rate, (2) classify indications, (3) delineate the pre-RCI clinical course, and (4) measure surgical and speech perception outcomes, in a large cohort of patients implanted in a tertiary referral center between 1989–2018. Retrospective data review was performed and included patient demographics, medical records, and audiologic outcomes. Results indicated that RCI rate was 11.7% (172/1465), with a trend of increased RCI load over the years. The main indications for RCI were device-related failures (soft-45.4%, hard-23.8%), medical failure (14%), trauma (8.1%), and surgical failure (6.4%). Success rate was 98.8%. Children comprised 78% (134) of the cohort and were more likely than adults to undergo RCI. Most (70%) of the RCIs were performed within 10 years from primary implantation. Speech perception outcome analysis revealed unchanged or improved performance in 85% of the cases and declined performance in 15%. Current findings confirm that RCI is a safe with high clinical efficacy; however, the non-negligible percentage of patients that exhibited declined performance post-RCI should be considered in decision-making processes regarding RCI. Routine follow-up during their first years post-implantation is warranted.

## 1. Introduction

The number of cochlear implantations (CIs) is continuously growing due to expanded candidacy criteria, technological advances, successful hearing outcomes, and persistent increase in life expectancy. As with any implanted device, there is an inherent risk for failure, infection, and rejection of the device consequently necessitating revision surgery [1,2]. Revision cochlear implantation (RCI) was initially reported in 1985 as a scarce event [3], however, since then the fraction of CIs that required revision surgery has considerably increased [4]. The overall RCI rates in the existing literature are widely variable, ranging from 1% [5] to 15.1% [4,6,7,8].

The indications for RCI were traditionally classified into hard, soft, surgical, and medical failures [1,9,10,11,12]. Hard and soft failures, categorized as device-related failures, have been claimed to be the leading indications for revision surgeries [1,12]. Hard failure is defined as an inability to present electric stimulation due to lack of communication between the internal and external hardware, resulting in diminished or lack of sound perception (usually sudden or rapid). Soft failure was defined in the 2005 Consensus Development Conference statement as an uncommon occurrence in which a device malfunction is suspected, but cannot be proven using currently available in vivo methods [13]. Clinically, soft failure presentation may include declined or unexpected poor performance, non-auditory aversive symptoms, and intermittent function. It is a diagnosis of exclusion [2,9,13,14], performed after all medical, imaging, and programing issues have been ruled out and external components have been replaced. Medical and surgical indications, categorized as non-device-related failures, include medical complications (infection, biofilm, allergic reaction) or surgical related reasons (malposition, electrode migration, or device extrusion) [2]. Although this widely used classification provides a clinical framework, indications were interpreted differently across RCI studies, suggesting that determination of RCI indication remains a challenging decision in some cases.

There is a general consensus that RCI is a safe surgery [15,16], which usually restores, or even improves, speech perception outcomes [12,14,17,18,19,20]. Several reports, however, have documented declined performance in 2.9–27.3% of the cases [10,15,17,21,22]. A main source of variability in speech perception outcomes may be attributed to different methods used to measure and define a significant change following RCI. While in most studies results were reported as group mean differences in performance before vs. after RCI [10,20], other authors applied a clinical criterion for comparing performance [15,17,18,23]. Nevertheless, even when a clinical criterion was applied to define change in performance, determination of whether the change was clinically significant was inconsistent across studies.

In view of the variability in RCI outcomes across studies, characterizing a large-scale RCI cohort may improve our understanding of the indications and outcomes, and consequently improve clinical decision making and patients’ counseling. The present study was designed, therefore, to characterize RCI at the Sheba Medical Center (SMC) program since its foundation, including (1) evaluation of RCI rate, (2) classification of indications for RCI, (3) delineation of the pre-RCI clinical course, and (4) measurement of surgical and speech perception outcomes.

## 2. Materials and Methods

A retrospective chart review was conducted for patients that underwent RCIs since the initiation of the CI program at the SMC in 1989 to the end of 2018. Data is reported for all RCI patient, including patients that had their primary CI (Pri-CI) in a different CI center and patients that were lost to follow up.

A revision case was defined as reimplantation surgery of the same ear as the Pri-CI, either primarily or in a delayed fashion. For each case, the following data were extracted:

(a) Relevant history, including demographic characteristics and the time-course of symptoms that led to RCI (specifically the duration between Pri-CI and symptoms onset as well as between symptoms onset and RCI).
(b) Reports of medical follow-up and surgical procedures, including imaging [computed tomography (CT), magnetic resonance imaging (MRI) or plain film Stenver’s view].
(c) RCI indication. A comprehensive case-by-case review by a panel of two senior audiologists and two neurotologists was performed to determine the following RCI indications: (a) Device-related indications—Soft failure was defined according to the 2005 consensus guidelines [13] and included cases with decreased or unexpected poor performance, non-auditory aversive symptoms, and intermittent function. In all of the soft failure cases, medical, imaging, programing, and hardware issues have been ruled out. Hard failure was determined according to the absence of communication between the internal and external hardware. (b) Non-device-related indications—Medical failure included cases with suspected biofilm infection, allergic response, neuralgia and chronic middle ear condition. Patients with severe congenital inner ear malformations (i.e., common cavity, hypoplastic nerve, others) were included in the medical indication group as well. Following a thorough case-by-case review, our assumption was that these patients’ medical status (reflected in severe inner ear anomalies) was the main reason for the symptoms (i.e., poor speech perception, extracochlear manifestations) that ultimately led to reimplantation. Surgical failure included malposition or inadequate electrode insertion, electrode migration or protrusion, and device extrusion. Trauma included implants that failed immediately after an event of head trauma.
(d) Speech perception was evaluated by means of an open-set monosyllabic word recognition test [Hebrew Arthur Boothroyd [24]; HAB] scored for correct words and phonemes. When the HAB test could not be administered due to young age, poor cognitive-linguistic skills or limited speech perception ability, speech reception threshold (SRT) was used. Both tests were administered in a quiet listening condition. First, we compared between speech perception scores measured before RCI (i.e., most recent measurement prior to appearance of symptoms), to those obtained 6 months or more after RCI. The difference score was calculated and subsequently the performance in each case was classified as unchanged, declined, or improved. The clinically significant criterion was defined as a change of >10% in HAB phoneme scores. Phoneme score, rather than word score, was selected as it was found to be less dependent on linguistic abilities, showed reduced variability and was therefore considered a more valid measure [25,26,27]. Analysis of HAB results of 90 adult CI recipients from our CI program (not included in the current study) revealed that between-lists phoneme score variability did not exceed 10%. Accordingly, the selected criterion for post RCI change was set to >10%. When phoneme scores were unobtainable, a change of ≥10 dB in SRT was used.

Statistical analysis. IBM SPSS Statistics version 22.0 software program (Armonk, NY: IBM Corp) was utilized. Mann–Whitney U and Chi-square tests were used to compare quantitative and categorial variables, respectively. Stepwise logistic regression was performed to study effects of predictor variables on speech perception outcomes. For all tests *p* < 0.05 was considered significant.

The study was conducted according to the guidelines of the Declaration of Helsinki, and approved by the Sheba Medical Center Institutional Review Board, SMC-16-3696.

## 3. Results

### 3.1. Demographics, RCI Rates and Time-Course

During the study period, 145 patients underwent 172 RCIs. These represent 14.8% and 11.7% of the total number of patients (976) and implantations (1465), respectively. Patients’ demographics are detailed in Table 1. No significant differences were found between gender nor RCI side. RCI among pediatric patients (<18 years at the time of Pri-CI) amounted to 18% of the total pediatric CI group (*n* = 629), and among adult patients amounted to 8.6% of the total adult group (*n* = 347). A significant association between age at Pri-CI and RCI was found, indicating that children were more likely than adults to undergo RCI (*X*^2^(1) = 12.5, *p* < 0.001, effect size Cramer’s *V* = 0.11). Our cohort included 18 (12.4%) patients who underwent more than one RCI in the same ear, 76.5% of them were children (at the time of Pri-CI). Thirteen RCIs (9%) were related to manufacturer recalls: 6 cases of Advanced Bionics Hires-90K recall (2006); 7 cases of Cochlear Nucleus 5 recall (2011).

**Figure 1:**
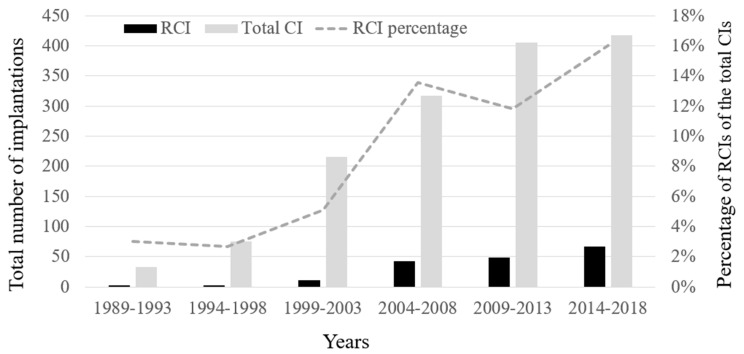
The burden of RCI of the total number of CIs 1989–2018. There was a gradual increase through the 30-year time-course in the proportion of revision cochlear implantation (dashed line). CI—cochlear implant; RCI—revision cochlear implant.

There was a gradual increase through the 30-year time-course in the proportion of RCI (Figure 1). The J-curve point of the RCI percentage, occurring in the years 2004–2008, represents a trend of sharp increase in the proportion of RCIs. During the first 15 years (1989–2003), the average rate of RCI was 4.3% (*n* = 14/324), whereas during the remaining 15 years (2004–2018) the rate increased to 13.8% (*n* = 158/1141).

**Figure 2:**
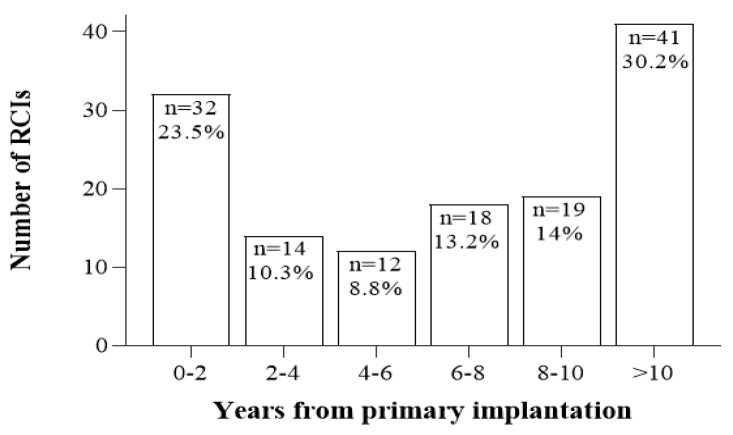
Time interval between primary cochlear implantation and revision surgery. Inclusion criteria for this analysis was cases with ≥ 10 years of follow-up (*n* = 136). The percentages refer to the number of cases included in this analysis. RCI—revision cochlear implant.

Three RCI time-course intervals were studied and are summarized in Table 2: time between (1) Pri-CI and RCI; (2) Pri-CI and onset of symptoms; (3) Onset of symptoms and RCI. Comparisons between children and adults revealed that the time between Pri-CI and onset of symptoms was approximately 9 months longer in children (*U*(170) = 1923, *p* = 0.03, effect size *r* = −0.17). Figure 2 presents the number of RCIs as a function of time from Pri-CI until revision. The highest risk for revision was found during the first 2 years (*n* = 32/136, 23.5%) and above 10 years (*n* = 41/136, 30.2%) after Pri-CI.

### 3.2. RCI Indications

**Figure 3:**
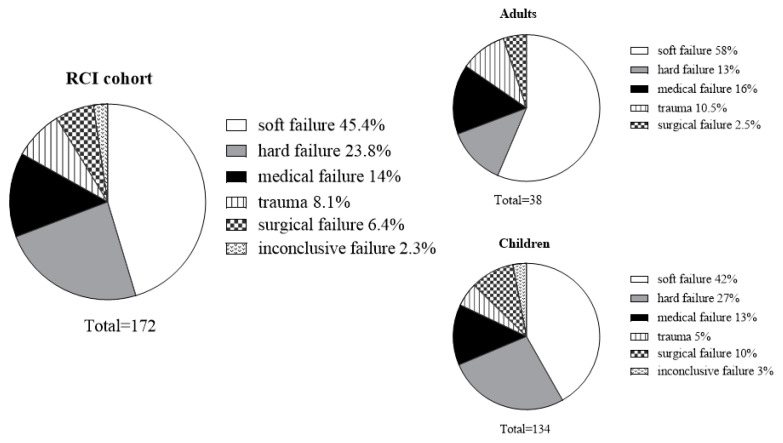
The distribution of revision cochlear implant (RCI) indications in all cases and divided by adults vs. children.

The leading indication for RCI was device-related soft and hard failures (Figure 3), accounting together for 69.2% of the cases in both children (*n* = 92 (69%): soft failure—42%, hard failure—27%) and adults (*n* = 27 (71%): soft failure—58%, hard failure—13%). Regarding non-device-related indications, medical failure accounted for 24 cases (14%) and was the leading indication in both age groups. The reasons for medical failure were congenital severe inner ear malformation (*n* = 8), suspected biofilm infection (*n* = 6), allergic response (*n* = 5), neuralgia (*n* = 4) and chronic suppurative otitis with severe tympanic-membrane retraction over the electrode (*n* = 1). Surgical failure accounted for 11 cases (6.4%), including: extra-cochlear insertion (*n* = 4), partial insertion (*n* = 2), electrode migration (*n* = 2), electrode protrusion through the ear drum (*n* = 2) and over-insertion (*n* = 1). Through the years 1999–2015 implantations in our program were performed either via mastoidectomy posterior tympanotomy approach (MPTA) or via suprameatal approach (SMA) [28]. Before and after this period implantations were performed via MPTA only. There was no difference in the surgical failure rate between the 2 approaches (SMA = 0.6%, 5/837, MPTA = 0.96%, 6/625). Trauma accounted for 14 cases (8.1%), and 4 cases (2.3%) were classified as inconclusive failures due to partial data. Although comparisons between adults and children by indication did not yield a significant difference (*X*^2^(4) = 7.1, *p* = 0.13), trauma was 4 times more common and hard failure was twice more common in children compared to adults (trauma 10% vs. 2.5%; hard failure 27% vs. 13% in children and adults, respectively).

### 3.3. Audiologic Outcomes

Speech perception data before and after RCI were available for 136 cases (79% of the 172 total cohort). Of these, 86 cases were classified according to the phoneme-based criterion and 50 cases according to the SRT-based criterion. In the remaining 36 cases (31% of the total cohort) speech perception data were not available due to young age at testing (*n* = 11), less than 6 months between Pri-CI and RCI or between Pri-CI and onset of symptoms (*n* = 15), missing data or lost to follow up (*n* = 10). Results indicated that speech perception following RCI was unchanged in 91 cases (67%), improved in 24 cases (18%), and declined in 21 cases (15%).

**Figure 4:**
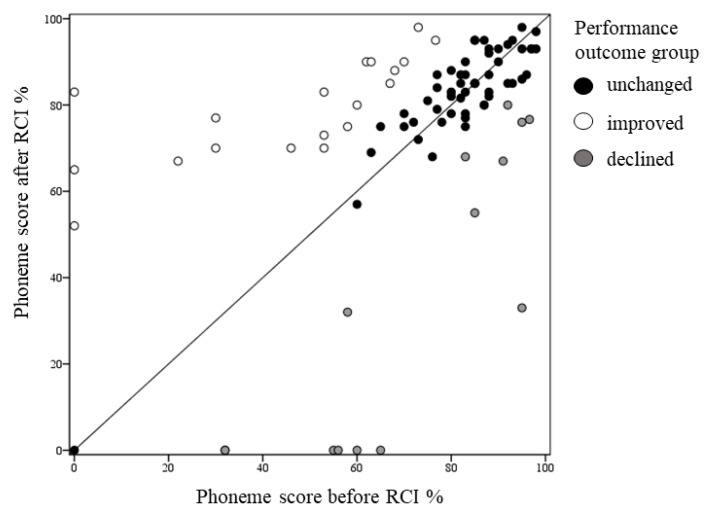
Individual phoneme scores before and after revision cochlear implantation (RCI), presented for each case (*n* = 86) according to performance outcome groups (unchanged, improved, declined). The diagonal line represents the same scores before and after RCI.

Figure 4 depicts individual phoneme scores (*n* = 86) before and after RCI for each case according to the performance outcome groups (improved, unchanged, declined). The mean phoneme score change for the improved performance group was 32.4% ± 18.4, for the unchanged performance group 0.5% ± 5.5 and for the declined performance group −37.6% ± 19.2. As can be seen, in seven cases phoneme scores declined below 40% following RCI. Detailed inspection revealed that: (1) In three cases (two patients) RCI indication was classified as medical due to severe inner ear malformations; (2) In one case, RCI was performed due to neuralgia-related pain, following a long period of non-use. Limited usage prior to RCI, together with poor motivation, may have impacted post-RCI performance in this case; (3) In the remaining three cases (two patients) the reason for declined performance could not be specified. Medical, surgical, and programming issues were ruled out, nonetheless, the patients failed to achieve the excepted improvement.

**Figure 5:**
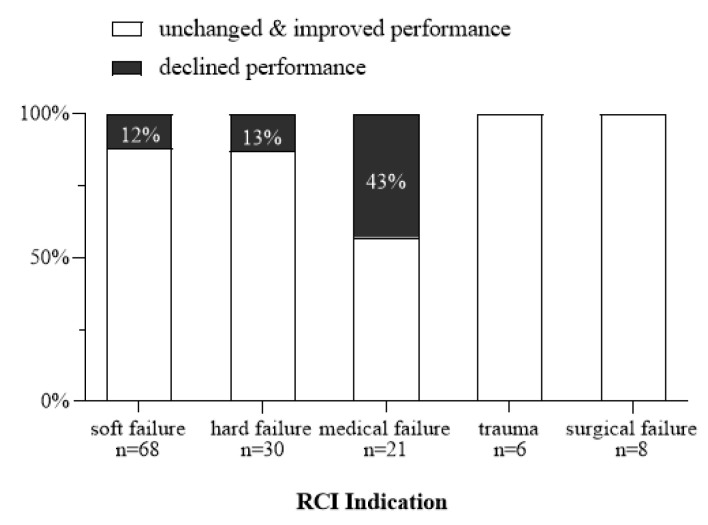
Distribution of performance outcome by revision cochlear implantation (RCI) indication.

Cases with declined performance were compared with those in which performance did not change or improved. Logistic regression analysis revealed that the independent variables: age, time from Pri-CI to RCI, and pre-RCI phoneme scores were not significant predictors of declined performance (declined vs. unchanged and improved, respectively: age (years)– mean 11 ± 16 vs. 13.5 ± 17.6, *β* = 1, *p* = 0.67; time (months) from PRI-CI to RCI– mean 132 ± 70.5 vs. 85 ± 64, *β* = 1, *p* = 0.1; pre-RCI phoneme score (%)– mean 69 ± 23 vs. 73 ± 24, *β* = 0.99, *p* = 0.6). As illustrated in Figure 5, however, a significant association was found between performance outcome groups (declined vs. unchanged and improved) and RCI indications (*X*^2^(10) = 19.6, *p* = 0.02, effect size Cramer’s *V* = 0.27). This finding may be explained by the higher percentage of declined performance in the medical indication group (43%) compared to the soft (12%) and hard (13%) failure indications. Additionally, none of the cases who underwent RCI for surgical and trauma indications showed declined performance.

### 3.4. Surgical Outcomes

Surgical success was achieved in 170 cases (98.8%). In two cases (1.2%) reinsertion was not achieved: In one the electrode was misplaced into a hypotympanic air cell. Second revision was successful. In the other soft tissue collapse occurred in the scala tympani. Uneventful implantation in the contralateral ear was performed. Partial insertion (>2 electrodes outside of the cochlea) occurred in six (3.5%) other adults cases, none with inner ear anomaly. None of these presented declined speech perception following RCI.

## 4. Discussion

Revision surgeries have become an inherent part of CI programs and are expected to increase as the number of CIs rises [29,30]. To support revision decision-making and thoughtful care planning, the current study aimed to characterize RCIs in a large cohort of 172 cases. We report a RCI rate of 11.7%, with a trend of an increasing load of RCIs over the 30 years of activity in our CI program. In accordance with previous studies, children comprised most of our RCI study cohort (79%) and were found more likely than adults to require a RCI [9,17,31]. Wang et al. [4] reviewed the reported RCI rates from 28 high volume CI programs and found that the rate ranged between 1.2% to 15.1%, with an average rate of 7.6%. Of note, almost one-third of these programs reported an RCI rate over 10%, which is comparable to our findings. One possible explanation for the variability of RCI rates across studies [4,5,6,32,33] may be related to the different methods for measuring and reporting RCI rates, which were discussed in depth by others [4,7].

O’Neill and Tolley [7] examined CI reliability by studying the reported rates of all-cause revision surgery using a pool of 30 clinical studies involving over 6300 pediatric patients. The data were transformed to a common time base to allow an evaluation of the events following implantation. They found that at 10 years post-implantation, almost 30% of children with unilateral implants are expected to undergo RCI. Although this finding considerably exceeded other reported revision rates [4,34], it illustrated the importance of interpreting results with respect to a relevant timeframe. According to our data, the mean time interval between Pri-CI and RCI was 6.6 years, comparable to results of other studies, which reported a mean length of device use before revision of 4.7–6 years [4,16,17,35]. Detailed examination for cases with a long follow-up period (≥10 years) revealed that 24% of the cases underwent RCI within the first 2 years after Pri-CI, 46% within a period of 2–10 years, and the remaining 30% after more than 10 years (Figure 2). In other words, 70% of RCIs were performed in cases having an implant lifetime of less than 10 years. Further analysis, focusing on the time between Pri-CI and the onset of symptoms that led to RCI, revealed a significant prolongation in children compared to adults, presumably related to the unique challenges involved in diagnosing CI failures in the pediatric population [34,36]. Taken together, our results strongly emphasize the need for close monitoring and special attention regarding follow-up of babies and young children, especially during their first years of implant use.

In our current cohort, as well as in previous reports [7,31], the overall leading RCI indication was device-related soft and hard failures, which together accounted for 69.2% of the cases (*n* = 119). The previously reported proportion of hard vs. soft failures ranged from equal [19] to 4 times higher prevalence of hard failures [4,8,29]. Results of the current study, however, indicated a lower rate of hard (23.8%) compared to soft failures (45.4%). This inconsistency could be partially attributed to the definition of inclusion for the soft failure indication. According to the soft failure’s consensus paper [13], reimplantation with subsequent alleviation of symptoms strongly supports the diagnosis of soft failure. Nevertheless, in 12% of the cases, who we classified as soft failures, speech perception declined following RCI. Kimura et al. [37] referred to these cases as ‘presumed soft failures’ and accordingly we suggest that the definition of the soft failure category should be broadened to include cases with soft failure symptomatology that do not regain their expected post-revision performance. Further explanation of our results could be related to the inclusion of trauma as an independent indication in our study, distinguishable from hard failure, whereas other studies included trauma in the hard failure category [9,31]. Hard failure, and especially trauma, were found more common in children presumably due to greater vulnerability of young children to falls and injuries [1,31,35]. Further analysis to support the relationship between young age and trauma revealed that the prevalence of revisions performed due to trauma steadily declined over the years post Pri-CI–from an average of 22% in the first two years post Pri-CI to 2% after 10 years. In accordance with their obvious clinical presentation, both trauma and hard failure were characterized by a significantly shorter time period between the onset of symptoms and RCI, compared to all other indications.

Monitoring and reporting speech perception measures pre- and post-revision are crucial to understand RCI functional outcomes. To the best of our knowledge, the current study presents speech perception results for the largest cohort reported so far, consisting of 136 cases. Utilizing a clinically meaningful speech perception criterion to evaluate change following RCI, we demonstrated unchanged or improved performance in the vast majority of our RCI cases (85%). Together with the 98.8% surgical success rate, these findings further support the safety and efficacy of RCI [10,16,17,21,37,38].

Declined performance was demonstrated in 15% (*n* = 21) of our cases. Although declined performance has a profound impact on patients’ communication skills and, consequently, on their quality of life, it has received minor attention in the literature and tends to be overlooked in revision studies. With regard to studies that applied a clinical criterion for evaluating a change in performance post-RCI, Reis et al. (2017) [17] as well as Mahtani et al. (2014) [18] used a criterion of ≥10% change in speech recognition scores for different sentence tests, and reported declined performance in 15.8% and 8% of the RCIs, respectively. Rivas et el. (2008) [15] used a criterion of 15% change for word or sentence tests and reported poorer performance in only 3% of the cases. In an attempt to characterize our cases with declined performance, we found that they could not be predicted by age, time from Pri-CI to RCI, or speech perception scores before RCI. Similar findings were reported by Mahtani et al. (2014) [18] regarding age and time. The most common indication related to declined performance was found to be medical failure, followed by hard and soft failure. Surgical failure and trauma were not related with declined performance. Possible explanations for this finding may be related to specific patients’ characteristics in the medical indication group (*n* = 24): (1) Relatively high percentage of severe inner ear malformations (8 cases, 33%), and (2) High incidence of patients presented with neuralgia- or biofilm-related pain (11 cases, 46%). These patients had long periods of limited CI usage prior to RCI, which could have impacted their speech perception outcomes. Further research is required to closely examine declined performance and its associated risk factors.

## 5. Conclusions

The aim of the present study was to examine the rates, indication and outcomes of a large RCI cohort (*n* = 172). An overall RCI rate of 11.7% was found, mainly due to device-related soft and hard failure indications. Our findings, representing one of the largest-scale analysis of speech perception outcomes according to a clinically based criteria, confirm that RCI is a safe procedure with high clinical efficacy. An important clinically relevant finding of performance decrement following revision was demonstrated in 15% of the RCI cases, necessitating further investigation.

Time course analysis revealed that RCI predominantly occurred within 10 years from initial implantation. Considering the finding of higher risk for RCI in children, together with growing numbers of pediatric recipients with extended CI periods, the fraction of RCIs in CI programs is anticipated to grow, increasing its institutional burden. A frequent medical and audiological follow-up, especially for pediatric recipients in their first years post-implantation, may benefit early identification of RCI symptoms and consequently improve case management.

## Figures and Tables

**Table 1:** Demographic variables.

	Total	Children	Adults
**Number of** (%)			
RCIs	172	134 (76%)	38 (24%)
patients	145	115 (79%)	30 (21%)
**Gender** (% †)			
female	69 (48%)	50 (43.5%)	19 (63%)
male	76 (52%)	65 (56.5%)	11 (37%)
**Side** (% ‡)			
right	80 (46.5%)	56 (42%)	24 (63%)
left	92 (53.5%)	78 (58%)	14 (37%)
**Age at primary CI** (years)			
*M* ± *SD*	12.5 ± 16.9	4.7 ± 3.8	39.9 ± 16.6
median (range)	4.5 (9 months-76.8)	3.3 (9 months-17.8)	34.7 (18.5–76.8)
**Approach of primary CI** (% ‡)			
SMA	115 (67%)	89 (66.4%)	26 (68.4%)
PTA	57 (33%)	45 (33.6%)	12 (32.6%)
**Hearing loss etiology** (% †)			
Genetic	57 (39.3%)	53 (46.1%)	4 (13.3%)
unknown	48 (33.1%)	35 (30.4%)	13 (43.3%)
inner ear malformation	10 (6.9%)	8 (7%)	2 (6.7%)
intrauterine infection		8 (7%)	1 (3.3%)
neonatal complication	7 (4.8%)	6 (5.2%)	1 (3.3%)
meningitis	3 (2.1%)	3 (2.6%)	0 (0%)
other	11 (7.6%)	2 (1.7%)	9 (30%)

CI—cochlear implantation; RCI—revision cochlear implantation; SMA—suprameatal approach; PTA—posterior tympanotomy approach; † percentage out of the number of patients; ‡ percentage out of the number of RCI cases.

**Table 2:** Time-course intervals, including comparison between children and adults.

Time-Course (Months)	Total	Children	Adults	Statistical Analysis Mann–Whitney Test
**Pri-CI to RCI**				
*M ± SD*	80 ± 70.5	80 ± 65.4	83 ± 86.8	*U*(172) = 2396 *p*-value = 0.58
*range*		1–248	0.5–346	
**Pri-CI to onset of symptoms**				
*M ± SD*	54 ± 62.1	56.5 ± 60.2	45 ± 68.2	*U*(170) = 1923 *p*-value = 0.03 * effect size *r* = −0.17
*range*		0–219	0–285	
**Onset of symptoms to RCI**				
*M ± SD*	26 ± 40.7	23.5 ± 35.8	38 ± 53.7	*U*(170) = 2000 *p*-value = 0.06
*range*		0.5–240	0.5–234	

CI—cochlear implantation; Pri-CI—primary cochlear implantation; RCI—revision cochlear implantation; * *p*-values considered significant.

## Data Availability

Data will be available upon request (see contact information of the corresponding author).
